# Psychometric characteristics of the Spanish version of instruments to measure neck pain disability

**DOI:** 10.1186/1471-2474-9-42

**Published:** 2008-04-09

**Authors:** Francisco M Kovacs, Joan Bagó, Ana Royuela, Jesús Seco, Sergio Giménez, Alfonso Muriel, Víctor Abraira, José Luis Martín, José Luis Peña, Mario Gestoso, Nicole Mufraggi, Montserrat Núñez, Josep Corcoll, Ignacio Gómez-Ochoa, Ma José Ramírez, Eva Calvo, Ma Dolores Castillo, David Martí, Salvador Fuster, Carmen Fernández, Nuria Gimeno, Alejandro Carballo, Álvaro Milán, Dolores Vázquez, Montserrat Cañellas, Ricardo Blanco, Pilar Brieva, Ma Trinidad Rueda, Luis Álvarez, María Teresa Gil del Real, Joaquín Ayerbe, Luis González, Leovigildo Ginel, Mariano Ortega, Miryam Bernal, Gonzalo Bolado, Anna Vidal, Ana Ausín, Domingo Ramón, María Antonia Mir, Miquel Tomás, Javier Zamora, Alejandra Cano

**Affiliations:** 1 Departamento Científico, Fundación Kovacs, Palma de Mallorca, Spain; 2 Unidad de Cirugía del Raquis, Hospital Vall d'Hebrón, Barcelona, Spain; 3 Unidad de Bioestadística Clínica, Hospital Ramón y Cajal, Madrid, Spain; 4 Departamento de Enfermería y Fisioterapia, Universidad de León, Ponferrada, Spain; 5 Centro de Salud del Limonar, Málaga, Spain; 6 Servicio de Medicina Preventiva, Hospital de Rehabilitación y Traumatología Virgen de las Nieves, Granada, Spain; 7 Servicio de Reumatología, Hospital Marqués de Valdecilla, Santander, Spain; 8 Clínica Kovacs, Fundación Kovacs, Palma de Mallorca, Spain; 9 Servicio de Cirugía Ortopédica y Traumatología, Hospital Clínico, Barcelona, Spain; 10 Centro de Salud de Tramuntana Esporlas, Mallorca, Spain; 11 Servicio de Rehabilitación, Hospital Lozano Blesa, Zaragoza, Spain; 12 Unidad de Rehabilitación, Hospital Marqués de Valdecilla, Santander, Spain; 13 Delegación Asuntos Sociales, Granada, Spain; 14 Servicio de Traumatología y Ortopedia, Hospital Parc Taulí de Sabadell, Barcelona, Spain; 15 Centro de Salud de Valldargent, Palma de Mallorca, Spain; 16 Servicio de Rehabilitación, Mutua Asepeyo, Madrid, Spain; 17 Servicio de Anestesia, Hospital Parc Taulí de Sabadell, Barcelona, Spain; 18 Servicio de Traumatología, Fundación Jiménez Díaz, Madrid, Spain; 19 Servicio de Neurocirugía, Fundación Jiménez Díaz, Madrid, Spain; 20 Centro de Salud Serrería II, Valencia, Spain; 21 Centro de Salud de Ciudad Jardín, Málaga, Spain; 22 Centro de Salud de Es Trencadors de Lluchmajor, Mallorca, Spain; 23 Servicio de Rehabilitación, Hospital Clínico Universitario, Valladolid, Spain; 24 Unidad Básica de Es Castell, Menorca, Spain; 25 Centro de Salud de Inca. Mallorca, Spain; 26 Servicio de Emergencias 061. Mallorca, Spain

## Abstract

**Background:**

The NDI, COM and NPQ are evaluation instruments for disability due to NP. There was no Spanish version of NDI or COM for which psychometric characteristics were known. The objectives of this study were to translate and culturally adapt the Spanish version of the Neck Disability Index Questionnaire (NDI), and the Core Outcome Measure (COM), to validate its use in Spanish speaking patients with non-specific neck pain (NP), and to compare their psychometric characteristics with those of the Spanish version of the Northwick Pain Questionnaire (NPQ).

**Methods:**

Translation/re-translation of the English versions of the NDI and the COM was done blindly and independently by a multidisciplinary team. The study was done in 9 primary care Centers and 12 specialty services from 9 regions in Spain, with 221 acute, subacute and chronic patients who visited their physician for NP: 54 in the pilot phase and 167 in the validation phase. Neck pain (VAS), referred pain (VAS), disability (NDI, COM and NPQ), catastrophizing (CSQ) and quality of life (SF-12) were measured on their first visit and 14 days later. Patients' self-assessment was used as the external criterion for pain and disability. In the pilot phase, patients' understanding of each item in the NDI and COM was assessed, and on day 1 test-retest reliability was estimated by giving a second NDI and COM in which the name of the questionnaires and the order of the items had been changed.

**Results:**

Comprehensibility of NDI and COM were good. *Minutes needed to fill out the questionnaires *[median, (P25, P75)]: NDI. 4 (2.2, 10.0), COM: 2.1 (1.0, 4.9). *Reliability*: [ICC, (95%CI)]: NDI: 0.88 (0.80, 0.93). COM: 0.85 (0.75,0.91). *Sensitivity to change*: Effect size for patients having worsened, not changed and improved between days 1 and 15, according to the external criterion for disability: NDI: -0.24, 0.15, 0.66; NPQ: -0.14, 0.06, 0.67; COM: 0.05, 0.19, 0.92. *Validity*: Results of NDI, NPQ and COM were consistent with the external criterion for disability, whereas only those from NDI were consistent with the one for pain. Correlations with VAS, CSQ and SF-12 were similar for NDI and NPQ (absolute values between 0.36 and 0.50 on day 1, between 0.38 and 0.70 on day 15), and slightly lower for COM (between 0.36 and 0.48 on day 1, and between 0.33 and 0.61 on day 15). Correlation between NDI and NPQ: r = 0.84 on day 1, r = 0.91 on day 15. Correlation between COM and NPQ: r = 0.63 on day 1, r = 0.71 on day 15.

**Conclusion:**

Although most psychometric characteristics of NDI, NPQ and COM are similar, those from the latter one are worse and its use may lead to patients' evolution seeming more positive than it actually is. NDI seems to be the best instrument for measuring NP-related disability, since its results are the most consistent with patient's assessment of their own clinical status and evolution. It takes two more minutes to answer the NDI than to answer the COM, but it can be reliably filled out by the patient without assistance.

**Trial Registration:**

Clinical Trials Register NCT00349544.

## Background

Mechanical, non-specific or common neck pain (NP) may have an impact on the functional status of the patient, interfering with basic activities such as sleeping or personal care, as well as on many work-related activities. In fact, NP is a common cause of disability and work absenteeism [1].

Although pain may lead to disability, those are two different dimensions that should be assessed separately [2]. In the research environment, reliable and valid instruments to measure NP-related disability are needed to assess the effect of treatment on that variable. In clinical practice, it is important to reliably measure disability since it influences a patient's quality of life, work absenteeism and personal and societal costs. Early monitoring and accurate follow-up of disability are also useful for identifying patients at higher risk for chronic disability and for deciding treatment goals and methods at any given time. In order to be recommended, instruments for measuring disability should be accurate and reliable. To be used in practice, these instruments should not reduce consultation time, i.e., they should be simple and easy to score by the physician, and easily understood by the patients, who can answer the questionnaires in the waiting room without assistance.

The Neck Disability Index (NDI) and the Northwich Park Questionnaire are two questionnaires for measuring NP-related disability [3,4]. Both questionnaires derive from the Oswestry Disability Index for measuring low back pain-related disability [5], and were designed to be filled out directly by the patient. They consist of 10 items reflecting activities of daily living or impairments that can be influenced by NP. For each dimension, six possible answers are provided. The patient must mark the answer that better describes his/her current status. Option 1 scores 0 points and represents no limitation for that particular activity, whereas option 6 scores 5 points and represents the maximum possible limitation (Appendix 1). Therefore, the maximum possible score is 50. However, results are usually given as the percentage of that maximum possible score, so the range from best to worst is 0–100.

Dimensions explored in the NPQ are neck pain intensity, interference of neck pain with sleep, interference with sleep of pins and needles or numbness in the arms at night, duration of symptoms, carrying objects, reading and watching television, working and/or doing housework, social activities, driving and a comparison of current state with the last time the questionnaire was completed [4]. Dimensions explored in the NDI are neck pain intensity, personal care (washing, dressing, etc.), lifting, reading, headache, concentration, work, driving, sleeping and recreation [3]. The NDI is one of the most used scales for measuring NP-related disability, and it has been successfully translated into French [6], Brazilian Portuguese [7], Korean [8], and Turkish [9]. Additionally, a modified Swedish version also exists [10].

The Core Outcome Measure (COM) was first proposed as a set of outcome measures for low back pain patients [11]. An adaptation for neck pain patients was developed later, and has been assessed in patients with common neck pain and in those with whiplash [12,13]. It includes the following dimensions: "severity of pain" (questions 1a -on neck pain- and 1b -on pain referred to the shoulder or arm-), "function" (question 2), "well-being" (question 3), "disability" (question 4), "absenteeism" (question 5) and "satisfaction" (question 6). Each item has 5 possible answers. Answers for items 3 and 6 are ordered from worst to best, while the rest is ordered from best to worst. (Appendix 2) The final score is the mean of the scores for each item, so to obtain it, the order of answers for items 3 and 6 must first be reversed. The final score ranges from 1.0 (best possible state) to 5.0 (worst possible state) [11-13].

However, only a modified Spanish version of the NPQ existed for measuring NP-related disability in Spanish speaking patients [14], and there was no Spanish version of the NDI or COM for which psychometric characteristics were known.

Therefore, the objectives of this study were: 1) To translate into Spanish and culturally adapt the NDI and COM, 2) to validate their use among Spanish neck pain patients, 3) to compare their psychometric characteristics with those of the NPQ.

## Methods

### Study design

The study was carried out in three phases: the first was translation into Spanish and cultural adaptation of the NDI and COM; the second was a pilot study to assess the comprehensibility and reproducibility of those Spanish versions; and the third was a validation study to determine their metric characteristics and to compare them with those of the NPQ.

### Translation phase

The same methods were followed separately for both the NDI and COM questionnaires. Each questionnaire was translated into Spanish by two different and independent native Spanish speakers, who had no medical knowledge and were both unaware of the purpose of the translation and of the fact that another translator was doing the same task. Both Spanish translations were then compared for inconsistencies. The two translations were then retranslated, also blindly and independently, into English by two native English speakers. Each of the English translations was then compared with the original English questionnaire and checked for inconsistencies.

The Spanish version of the questionnaire was then separately reviewed and fine tuned by a bilingual team including the four translators, eight primary care physicians, four back specialists, and three methodologists (see Additional files 1 and 2).

### Pilot phase

The pilot phase was performed in 15 Centers located in 7 different administrative regions, of the 17 existing in Spain. All the Centers belong to the Spanish National Health System and are involved in the Spanish Back Pain Research Network. Participating Centers included 8 primary care centers and 7 hospital outpatient clinics in orthopedic surgery, rheumatology and rehabilitation.

The pilot study was carried out with patients who consulted their physician for NP between Oct 7, 2005, and April 5, 2006. Inclusion criteria were consulting for NP, with or without referred pain, being able to read Spanish and signing the corresponding written informed consent. The study was approved by the Ethics Commission of the Hospital Parc Tauli (Sabadell, Barcelona) on Oct 5^th^, 2005.

Exclusion criteria were: functional illiteracy (mental status insufficient to be able to complete the questionnaires), treated or untreated central nervous system impairment, direct trauma to the neck, and criteria for referral to surgery or for suspecting a potential systemic disease. Criteria for referral to surgery were defined as clinically relevant motor weakness or disabling pain radiating down the arm for at least 6 weeks in spite of conservative treatment, caused by a nerve root compression demonstrated by magnetic resonance (MRI) or computed tomography (CT) studies. Reasons for suspecting a potential underlying systemic disease were defined as oncologic disease during the previous 5 years, constitutional symptoms -unexplained weight loss, fever, chills-, history of intravenous drug use, or immunocompromised host.

The sample size of the pilot study was established at 50 patients. According to the available evidence on low back pain patients, the limit between acute and subacute pain was established at 14 days [2,15], and the limit between subacute and chronic at 90 days.^16^

Patients were seen the day of admission to the study (day 1) and 14 days later (day 15). At the first visit, the following variables were recorded on the data collection form: sex, age, socioeconomic level, family situation, academic level, work status, duration of the current work status, chronicity of pain (defined as acute, subacute or chronic) [2,15,16], patients' subjective assessments of severity of pain (no pain, mild, moderate, severe, unbearable) and of degree of limitations in daily living due to neck pain (none, mildly limited, moderately, severely, or very severely limited). Those patients' subjective assessments were considered as the "external criterion" for severity of pain and disability, respectively.

In addition, diagnostic procedures and treatments that patients had undergone were recorded, and those subjects in whom cervical disc herniations had been observed on MRI or CT scans were identified (Table 1).

**Table 1:** Characteristics of study participants.

Variable; n(%)	N (%)
	N = 221
Gender:	
Female	172 (77.8)
Male	49 (22.2)
	
Age; median (SD)	48.7 (14.8)
	
Recruitment setting	
Primary care	43 (19.5)
Specialized care	178(80.5)
	
Duration of current episode (days); median (P25;P75)	40.0 (15.0; 90.0)
	
Chronicity	
Acute (1–14 days)	46 (20.8)
Subacute (14–90 days)	152 (68.8)
Chronic (> 90 days)	22 (10.0)
Missing	1 (0.5)
	
Family situation	
Single	48 (21.7)
Married	131 (59.3)
Widowed	19 (8.6)
Divorced	21 (9.5)
Other	1 (0.5)
Missing	1 (0.5)
	
Academic level	
Less than elementary school	21 (9.5)
Elementary school	81 (36.7)
High school	74 (33.5)
University	39 (17.6)
Missing	6 (2.7)
	
Working situation	
Self-employed	23 (10.4)
Employed	121 (54.4)
Retired	34 (15.4)
Without work	6 (2.7)
Student	5 (2.3)
Housewife	24 (10.9)
Missing	8 (3.6)
	
External criterion for neck pain	
No pain	7 (3.2)
Mild pain	74 (33.5)
Moderate pain	83 (37.6)
Severe pain	49 (22.2)
Very severe pain	8 (3.6)
	
External criterion for neck disability	
None	7 (3.2)
Mild	56 (25.3)
Moderate	82 (37.1)
Severe	59 (26.7)
Very severe	16 (7.2)
Missing	1 (0.5)
	
X-rays	
No	105 (47.5)
Yes	116 (52.5)
	
CT Scan	
No	206 (93.2)
Yes	15 (6.8)
	
MRI	
No	176 (79.6)
Yes	45 (20.4)
	
Image of cervical disc herniation	
No	177 (80.1)
Yes	42 (19.0)
Missing	2 (0.9)
	
Electromyography	
No	210 (95.0)
Yes	11 (5.0)
	
Drug Treatment	
No	29 (13.1)
Yes	192 (86.9)
	
Education (traditional biomechanic)	
No	199 (90.0)
Yes	21 (9.5)
Missing	1 (0.5)
	
Education (active management)	
No	214 (96.8)
Yes	7 (3.2)
	
Physiotherapy (passive)	
No	182 (82.4)
Yes	39 (17.6)
	
Exercise	
No	196 (88.7)
Yes	25 (11.3)
	
Rehabilitation	
No	208 (94.1)
Yes	13 (5.9)
	
NRT intervention	
No	195 (88.2)
Yes	26 (11.8)
	
Epidural injections	
No	219 (99.1)
Yes	2 (0.9)
	
Facet joint denervation	
No	216 (97.7)
Yes	5 (2.3)
	
Surgery	
No	219 (99.1)
Yes	2 (0.9)
	
Other treatments	
No	204 (92.3)
Yes	17 (7.7)

At both visits, patients were given two separate Visual Analogue Scales (VAS) [17] for measuring neck pain and pain referred to the arm, the NDI, COM and NPQ questionnaires to assess neck pain-related disability, the previously validated Spanish versions of the SF-12 questionnaire for measuring general quality of life [18], and the Coping Strategies Questionnaire (CSQ) [19,20] to assess catastrophizing thoughts. VAS values range from better to worse, from 0 to 10, and CSQ from 0 to 36 [17,19,20]. Within the SF-12 two subscales are defined: the physical component summary (PCS-SF12) and the mental component summary (MCS-SF12). Higher scores reflect better quality of life, and values have been normalized so that mean values on both subscales for the Spanish population are 50, and SD is 10. Values range from 19.85 to 56.71 for PCS-SF12, and from 14.15 to 68.45 for MCS-SF12 [18].

All self-assessment questionnaires were given by administrative staff and the patients filled them out on their own and alone, without the presence of staff or accompanying persons. Requests for aid in interpretation of the items in the NDI and COM questionnaires were registered. The completed instruments were then given to the treating physician, who stapled scales and questionnaires onto the patient's data collection form.

Patients were told that several questionnaires were going to be given, and were asked to notify the staff in case that any of them was given twice. On day 1, each patient was given a first NDI and COM questionnaire. The time needed for answering each one was recorded. To assess repeatability, patients were asked to fill out the VAS, RMQ and SF12, and at least 30 min. after having answered the NDI and COM the patient was given a second version of those questionnaires. Questionnaires in this second set were printed in differently colored paper, listed the items in a different order and were not titled "NDI" and "COM", but "NID" and "CSC6". Finally, the clinician filled out a standardized questionnaire asking each patient about his or her interpretation of the meaning of each of the items in the NDI and the COM.

It was decided that sentences for which more than 10% of patients in the pilot study needed clarification or misinterpreted the meaning would be reviewed before undertaking the validation study. Such review would be made by the bilingual team that developed the first version, based on the patients' suggestions and on the comments from the clinicians administering the questionnaire and interviewing the patients. It was also decided that if that team felt that potential modifications in the questionnaire were relevant enough, data gathered from patients included in the pilot phase would not be used for the objectives of the validation phase.

Data were entered in the database at a centralized coordination office. Entry of data was done independently by two administrative assistants, who double-checked that the data they were entering coincided with the scores of the two VAS scales and the NPQ, NDI, NID, COM, CSC6, CSQ, and SF-12 questionnaires.

### Validation phase

The validation phase was performed in 12 Health Care Centers from 7 different regions in Spain, including two regions from which no Center had participated in the pilot phase. Ten Centers belong to the Spanish National Health System (SNHS) and two to not-for-profit Foundations working for SNHS. All the Centers are involved in the Spanish Back Pain Research Network. Participating Centers included 3 primary care centers and 9 specialty centers in rehabilitation, neuroreflexotherapy, orthopedic surgery, rheumatology and neurosurgery, five of which did not participate in the pilot phase.

The validation study was carried out with subjects who consulted for neck pain between April 6, 2006 and Feb 1, 2007. In order to ensure a sufficient number of acute, subacute and chronic patients, the sample size was established at 150 with a minimum of 15 in each of the three subgroups (acute, subacute and chronic). The only differences with methods used in the pilot phase were: 1) the time needed to fill out the NDI and COM was not registered, 2) only one version of the questionnaires was given (NID and CSC6 were not used), and 3) patients were not asked about their comprehension of each item in the questionnaires.

### Analysis

Comprehension was determined in the pilot study by the patients' answers to the questions exploring their understanding of each item on the NDI and COM questionnaires, and was measured in both the pilot and validation studies by the patients' requests for aid in interpretation and by the number of items which were not answered in each questionnaire.

The distribution of answers across categories was assessed for each item, and potential ceiling and floor effects were estimated by calculating the percentage of subjects indicating the maximum and minimum possible scores for the NDI, COM and NPQ questionnaires.

Sensitivity to change was estimated by calculating the effect size of NDI, NPQ and COM in patients that, according to external criteria for pain and disability, had worsened, not changed or improved between days 1 and 15. Worsening and improvement in pain and disability were defined as any negative or positive change in the corresponding external criterion. For each questionnaire, effect size was calculated as the difference between scores on day 1 and 15, divided by the standard deviation of the score on day 1. According to this method, an effect size < 0.20 corresponds to no change, 0.20–0.49 to a small change, 0.50 to 0.79 to a moderate change and ≥ 0.80 to a great change [21-23].

Test-retest reliability was measured in the pilot phase, comparing the results of the first and second NDIs, identified respectively as "NDI" and "NID", and the results of the first and second COM, identified respectively as "COM" and "CSC6". Reliability was assessed through the kappa index for answers given to the same items in both versions of each questionnaire. The reliability of the total score was assessed through the intraclass correlation coefficient [24] and the Bland-Altman method [25]. In addition, the total scores of both versions of the NDI were classified as reflecting "no disability" (NDI < 10% of maximum total score), or a "mild" (NDI between 10% and < 30%), "moderate" (NDI between 30% and < 50%), "severe" (NDI between 50% and < 70%) or "very severe" (NDI >= 70%) degree of disability [2]. The kappa index was used to compare those total scores. To that end, bi-square weights [26] were used. Since results from the COM are not categorized, this approach was only used for NDI

Cronbach's alpha was used to evaluate internal consistency of the NDI and NPQ [27]. Since COM aggregates several subscales, Cronbach's alfa was calculated only for the subscales on pain and disability of that questionnaire. Validity was measured by Spearman's correlation coefficients between VAS, CSQ, PCS-SF12, MCS-SF12, NPQ, NDI and COM values, for days 1 and 15 [17]. In addition, median (P25, P75) total scores of NDI, COM and NPQ were calculated for each category in the external criteria for pain severity and disability.

## Results

A total of 221 patients were eligible and none were excluded. Fifty-four patients were recruited for the pilot study and 167 for the validation study. Forty-two (19.0%) showed images of cervical disc herniation on MRI. For the pilot study, 23 patients were recruited from primary care centers and 31 from the hospital setting. For the validation study, 20 patients were recruited from primary care centers and 147 from the hospital setting (Table 1).

Table 1 shows the characteristics of the study subjects and Table 2 shows values for scores on the VAS, NDI, COM, NPQ and SF-12 for days 1 and 15. Since data are slightly skewed, they are given as a median (P25, P75).

**Table 2:** Values for VAS, NDI, NPQ, COM, CSQ, and SF-12*

	Day 1		Day 15	
	n	Value	n	Value

Neck pain (VAS), range 0–10	221	6.0 (4.0; 7.0)	221	5.0 (3.0; 7.0)
Referred pain (VAS), range 0–10	221	4.0 (0.0; 7.0)	221	3.0 (0.0; 6.0)
Disability (NDI), range 0–100	221	37.5 (26.0; 51.1)	221	31.1 (20.0; 44.4)
Disability (NPQ), range 0–100	221	38.9 (30.5; 54.6)	221	34.4 (20.6; 48.6)
Disability (COM), range 1–5	217	2.7 (2.4; 3.4)	215	2.4 (1.9; 3.0)
Catastrophizing (CSQ), range 0–36	218	12.0 (3.0; 18.0)	219	7.0 (0.0; 15.0)
Physical quality of life (PCSF-12), range 19.85 to 56.71	215	35.4 (30.6; 43.0)	214	39.0 (33.2; 48.0)
Mental quality of life (MCSF-12), range 14.15 to 68.45	215	41.6 (29.7; 54.6)	214	45.7 (31.1; 55.0)

*: Data given as median (P25, P75)

The time needed to fill out the Spanish version of the NDI was 4 minutes (P25, P75: 2.2, 10.0), and for the Spanish version of the COM, it was 2.1 min. (1.0, 4.9).

In relation to the NDI, at the end of the pilot phase 10 patients had asked for aid in the interpretation of questions No. 3 (4 patients, 7.4% of those participating in the pilot phase), No. 8 (3 patients, 5.5%), No. 1 (2 patients, 3.7%), No. 2 (2 patients, 3.7%) No. 7 (1 patient, 1.9%) and No. 9 (1 patient, 1.9%), with 4 patients asking for help with two questions. In addition, 8 patients did not answer question No. 8 because they did not drive. Only four patients misunderstood the meaning of the following questions: N° 4 (2 patients, 3.7%), No. 5 (1 patient, 1.9%) and No. 7 (1 patient, 1.9%).

In relation to the COM, 10 patients asked for aid in interpretation of questions N° 6 (4 patients, 7.4%), No. 5 (4 patients, 7.4%), No. 4 (3 patients, 5.5%), No. 3 (2 patients, 3.7%), No. 2 (2 patients, 3.7%), No. 1b (1 patient, 1.9%) and No. 1a (1 patient, 1.9%), with one patient requesting help with 4 questions, one with 3 questions and three with 2 questions. Only one patient (1.9%) misunderstood the meaning of question No. 6. Therefore, the wording of the NDI and COM remained unchanged for the validation phase.

No patient notified the staff of having identified the NDI and the NID questionnaires, or the COM and the CSC6, as being the same. Cronbach's alfa was 0.89 for the NDI, 0.91 for the NID, 0.84 for the NPQ, 0.73 and 0.84 for the pain and disability subscales of COM, respectively, and 0.62 and 0.90 for the pain and disability subscales of CSC6, respectively.

In relation to the NDI, a comparison of the scores of both versions of the questionnaire yielded the following results [median (P25; P75)]: NDI: 36.9 (25.6; 51.8), NID: 35.0 (25.5; 46.2), with 68.5% of answers being identical in both questionnaires, and an intraclass correlation coefficient for both of 0.88 (95% IC; 0.80, 0.93). The limits of agreement between NDI and NID were 1.25 ± 18.33 (see Additional file 3). The mean of bi-square weighted kappa values for all items was 0.84. Six items had a substantial concordance of 0.61–0.80 (Nos. 3, 4, 6, 7, 8 and 10), and four (Nos. 1, 2, 5 and 9) an almost perfect concordance greater than 0.80 [28].

In relation to the COM, a comparison of the scores of both versions of the questionnaire yielded the following results [median (P25; P75)]: COM: 3.0 (2.6; 3.5), CSC6: 2.8 (2.6; 3.4), with 76.4% of answers being identical in both questionnaires, and an intraclass correlation coefficient for both of 0.85 (95% IC; 0.75, 0.91). One item had a moderate concordance of 0.54 (No. 6), two a substantial concordance of between 0.61 and 0.80 (Nos. 1a and 3), and four (Nos. 1b, 2, 4 and 5) an almost perfect concordance greater than 0.80 [28]. The limits of agreement between COM and CSC6 were 0.04 ± 0.76 (see Additional file 3).

All of the items of NDI, NPQ and COM had answers distributed across all categories. For the NDI, the lowest observed score was 4% (rated by 1 patient, 0.5% of the 221 subjects participating in the study), and the highest one was 86% (rated by 1 patient, 0.5%). For the COM, the lowest observed score was 1.2 points (rated by 2 patients, 0.9%) and the highest one was 5.0 (1 patients, 0.5%). For the NPQ, the lowest observed score was 5.6% (1 patient, 0.5%), and the highest was 84.4% (1 patient, 0.5%) (Table 3).

**Table 3:** Maximum and Minimum Scores: Floor and Ceiling Effects.

	Minimum Score Recorded	Patients With Minimum Score (Floor Effect)	Maximum Score Recorded	Patients With Maximum Score (Ceiling Effect)
NDI	4.0	1 (0.5%)	86.0	1 (0.5%)
NPQ	5.6	1 (0.5%)	84.4	1 (0.5%)
COM	1.2	2 (0.9%)	5.0	1 (0.5%)

NDI and NPQ scores are given as % (range 0–100). COM scores are given as points (range 1–5).

Results of NDI, NPQ and COM were consistent with the external criterion for disability, so that values for those questionnaires were higher as patient's self-perception of disability increased (Table 5). However, only results of the NDI were consistent with the external criterion for pain (Table 4). For NPQ, values were identical for subjects in the categories "severe pain" and "very severe pain". For COM, values were identical for patients in the "mild pain" and "moderate pain" categories, and were higher for those in the "severe pain" category than for those in the "very severe pain" category (Table 4).

**Table 4:** Values of NDI, NPQ and COM across categories for external criteria for pain*

External criterion for pain*	No pain	Mild pain	Moderate pain	Severe pain	Very severe pain
N	7	74	83	49	8
NDI	22.0 (18.0; 37.8)	30.0 (23.5; 38.0)	36.0 (26.7; 46.0)	56.0 (43.1; 69.4)	61.0 (51.7; 79.0)
NPQ	22.2 (11.1; 33.3)	33.3 (25.0; 41.7)	38.9 (32.1; 50.0)	55.5 (42.7; 69.1)	55.5 (44.6; 74.1)
COM	2.0 (1.6; 3.1)	2.6 (2.2; 3.0)	2.6 (2.4; 3.1)	3.4 (2.7; 3.8)	3.2 (3.0; 3.7)

*: Values are given as median (P25, P75). NDI and NPQ scores are given as % (range 0–100). COM scores are given as points (range 1–5).

**Table 5:** Values of NDI, NPQ and COM across categories for external criteria for disability*

External criterion for disability*	No disability	Mild	Moderate	Severe	Very severe
N	7	56	82	59	16
NDI	18.0 (12.0; 46.0)	27.3 (20.0; 35.5)	40.0 (28.0; 47.0)	44.0 (30.0; 60.0)	70.5 (58.5; 76.0)
NPQ	22.2 (13.9; 41.7)	30.5 (25.0; 37.1)	38.9 (33.3; 53.1)	47.2 (38.9; 62.5)	69.0 (54.8; 77.1)
COM	2.4 (1.6; 2.6)	2.5 (2.0; 2.9)	2.7 (2.4; 3.2)	3.0 (2.6; 3.7)	3.6 (3.1; 4.0)

*: Values are given as median (P25, P75). NDI and NPQ scores are given as % (range 0–100). COM scores are given as points (range 1–5).

Effect size of NDI, NPQ and COM for patients having worsened, not changed or improved according to external criteria for pain and disability are shown in Tables 7 and 8. As seen in those tables, the effect sizes of NDI and NPQ are consistent in showing moderate improvements in patients having actually improved according to the external criterion, while the effect size of COM magnifies the amount of that improvement. As to patients reporting to have actually worsened, the effect size of NDI shows a small change consistent with that external criterion, while the one of NPQ is close to the cut-off point for small change and the COM does not detect any change. The same trends are observed for results on pain (Table 6).

**Table 6:** Effect size of NDI, NPQ and COM for patients having worsened, not changed or improved between days 1 and 16, according to external criteria for pain and disability.

Scale	**Patients' status according to external criterion for disability**	N*	Mean	SD	Effect Size**
NDI	Worsened	29	-4.34	17.98	**-0.24**
	Unchanged	80	2.46	16.07	**0.15**
	Improved	111	12.20	18.52	**0.66**
NPQ	Worsened	29	-2.58	18.13	**-0.14**
	Unchanged	80	1.02	16.18	**0.06**
	Improved	111	11.69	17.51	**0.67**
COM	Worsened	26	0.04	0.89	**0.05**
	Unchanged	79	0.13	0.70	**0.19**
	Improved	106	0.63	0.69	**0.92**

Scale	**Patients' status according to external criterion for pain**	N*	Mean	SD	Effect Size**

NDI	Worsened	35	-4.05	16.21	**-0.25**
	Unchanged	82	4.02	17.54	**0.13**
	Improved	103	12.01	17.96	**0.67**
NPQ	Worsened	35	-5.17	19.36	**-0.27**
	Unchanged	82	3.21	17.02	**0.19**
	Improved	103	11.86	16.57	**0.72**
COM	Worsened	32	-0.02	0.75	**-0.02**
	Unchanged	78	0.25	0.66	**0.38**
	Improved	101	0.60	0.75	**0.79**

*: Total number of patients participating in the study was 221. Missing patients in these tables are due to missing values in either the questionnaire (NDI, NPQ or COM) or the external criterion for pain or disability

** Absolute values for effect size should be interpreted as follows: values < 0.20 correspond to no change, 0.20–0.49 to a small change, 0.50 to 0.79 to a moderate change and ≥ 0.80 to a great change.^21–23^

**Table 7:** Spearman Correlation Coefficients between NDI, COM, NPQ, VAS, CSQ, and SF-12 (Day 1).

	VAS (referred pain)	CSQ	NDI	NPQ	Physical Component Summary (PCS-12)	Mental Component Summary (MCS-12)	COM
VAS (NP)	**0.485**	**0.403**	**0.480**	**0.471**	**-0.246**	**-0.221**	**0.356**
P	0.000	0.000	0.000	0.000	0.000	0.001	0.000
N	221	218	221	221	215	215	217
VAS (referred pain)		**0.392**	**0.358**	**0.442**	**-0.431**	**-0.081**	**0.459**
P		0.000	0.000	0.000	0.000	0.238	0.000
N		218	221	221	215	215	217
CSQ			**0.482**	**0.452**	**-0.302**	**-0.369**	**0.369**
P			0.000	0.000	0.000	0.000	0.000
N			218	218	214	214	214
NDI				**0.844**	**-0.404**	**-0.498**	**0.610**
P				0.000	0.000	0.000	0.000
N				221	215	215	217
NPQ					**-0.458**	**-0.402**	**0.628**
P					0.000	0.000	0.000
N					215	215	217
Physical Component Summary (PCS-12)						**-0.118**	**-0.477**
P						0.085	0.000
N						215	212
Mental Component Summary (MCS-12)							**-0.379**
P							0.000
N							212

**Table 8:** Spearman Correlation Coefficients between NDI0, COM, NPQ, VAS, CSQ, and SF-12 (Day 15).

	VAS (referred pain)	CSQ	NDI	NPQ	Physical Component Summary (PCS-12)	Mental Component Summary (MCS-12)	COM
VAS (NP)	**0.519**	**0.584**	**0.697**	**0.699**	**-0.458**	**-0.274**	**0.610**
P	0.000	0.000	0.000	0.000	0.000	0.000	0.000
n	221	219	221	221	214	214	215
VAS (referred pain)		**0.384**	**0.468**	**0.489**	**-0.427**	**-0.170**	**0.458**
P		0.000	0.000	0.000	0.000	0.013	0.000
n		219	221	221	214	214	215
CSQ			**0.667**	**0.669**	**-0.536**	**-0.357**	**0.567**
P			0.000	0.000	0.000	0.000	0.000
n			219	219	212	212	213
NDI				**0.908**	**-0.574**	**-0.409**	**0.693**
P				0.000	0.000	0.000	0.000
n				221	214	214	215
NPQ					**-0.611**	**-0.380**	**0.706**
P					0.000	0.000	0.000
n					214	214	215
Physical Component Summary (PCS-12)						**-0.046**	**-0.594**
P						0.503	0.000
n						214	210
Mental Component Summary (MCS-12)							**-0.326**
P							0.000
n							210

Tables 7 and 8 show correlation among the scores of the VAS for NP, VAS for referred pain, NDI, COM and NPQ, CSQ, and SF-12 (Physical and Mental). As seen in those tables, on days 1 and 15 correlations among NDI, NPQ, COM, VAS, CSQ. PCS-SF12 and MCS-SF12 (Physical and Mental) were significant at the p < 0.001 level, except for the one between MCS-SF12 and VAS for referred pain on day 1, and MCS-SF12 and PCS-SF12 on days 1 and 15. Correlations of NDI and NPQ with the rest of the scales were similar and consistently stronger than correlations of COM. Correlations between NDI and NPQ were 0.84 on day 1 and 0.91 on day 15, whereas correlations between COM and NPQ were 0.63 on day 1 and 0.71 on day 15.

## Discussion

Results from this study show that the Spanish versions of both NDI and COM are comprehensible and appropriate instruments. In addition, they show that NDI, NPQ and COM are internally consistent and valid instruments to measure neck pain patients' disability, that floor and ceiling effects are not a major concern for any of those questionnaires and that they can be used in routine clinical conditions. In fact, this study was performed in routine conditions, no patient left the NDI and NPQ questionnaires unanswered, and only 4 out of 221 (1.8%) left the COM unanswered (Table 2).

According to results from this study, NDI is more effective than NPQ and COM to assess neck pain disability. It is reliable and shows the highest correlations with results from instruments to measure pain, disability and quality of life. In addition, it is the only questionnaire for which the evolution of its score is consistent with external criteria for pain and disability (Tables 4 and 5) and for which effect sizes for pain and disability are consistent with patients' assessment of their own clinical evolution (Table 6). According to these results, NPQ is the second best and COM is the worst. NPQ does not detect worsening in disability and it suggests pain improvement in patients denying such an improvement (Table 6). Although internal validity is similar for all the questionnaires and differences in correlation and reliability are small, COM is less reliable than NDI, and its correlations with all the other scales and questionnaires are lower than those for both NDI and NPQ. In addition, COM is insensitive to worsening for both pain and disability, it reflects improvement in pain for patients denying any change, and it magnifies the amount of improvement for pain and, especially, disability (Table 6). This implies that using the COM may lead to the evolution of patients appearing to be more positive than it actually is. The inferiority of COM to assess pain and disability may be due to its global score being influenced by patients' assessment of function, well being, absenteeism and satisfaction, as opposed to the scores of NDI and NPQ, which only focus on pain and disability.

Filling out the NDI requires two minutes more than answering the COM. However, both questionnaires can be appropriately filled out by the patient in the waiting room without assistance, so this aspect is not a major shortcoming for its use in routine practice. The time needed to score the questionnaires was not measured in this study, but physicians' feeling is that it is roughly similar for all of the questionnaires: NDI and NPQ are longer but scoring the COM is more complex, since it requires the reversal of the order of answers to questions No. 3 and 6, and to calculate the mean value of the answers to the 7 items in order to get the final score.

Those characteristics may help to select the questionnaire that is most suitable for use in a particular setting. Whenever possible, the NDI seems to be the best option, especially in research settings where reliability, validity, sensitivity to changes and getting results that match actual patients' perceptions are essential concerns. In addition, the NDI is already available in several languages [6-10], and considering one questionnaire as an international standard could boost the implementation of disability assessment of neck pain patients as a routine procedure in clinical practice, and would help to compare results in studies conducted in different settings. However, since it might be better to use the COM than not to assess NP-related disability at all, this questionnaire might also be an option to consider in clinical environments where saving two minutes in the waiting room may make a difference. However, users of the COM should be aware that the results they will get are likely to overestimate patients' improvement and may not detect actual worsening.

Reliability was measured in the pilot study on the same day, by giving the patient two different versions of the NDI and COM questionnaires. The interval after which the second version is to be given is a relevant decision; a too long interval may underestimate reliability by allowing actual changes in patients' degree of disability to occur, while a too short interval may overestimate it because of recall bias. At the design phase, it was decided to give both versions on the same day, and to implement measures to prevent recall bias. To that end, an interval of at least 30 minutes lapsed between both tests, and the patients were asked to fill out the VAS, NPQ, CSQ and SF-12 questionnaires in the meantime. In addition, the second version of both questionnaires had a different name at the top ("NID" instead of "NDI", "CSC6" instead of "COM"), the first version was taken once answered and before handing out the second one, and both versions listed the questions in a different order. Although the change in the order of the questions might alter the results, because a patient may consider a previous question when answering the next, it was felt that this risk was worthwhile in order to avoid recall bias. This method for testing reliability had previously proven feasible and valid in our environment [29-31]. In fact, none of the patients identified the NDI and the NID, or the COM and the CSC6, as being the same, suggesting that the measures undertaken to avoid recall bias worked well. In addition, in spite of the potential effect of the different order of the questions in the NID and CSC6, intraclass correlation coefficients, kappa values and results from the Bland-Altman method showed a good reliability for NDI and COM. Therefore, the reliability of these questionnaires should not be a concern.

In some previous studies, patients' subjective classification of their clinical evolution during the study period has been used as the external criterion [32-36]. That approach makes sense in studies where patients' subjective perception of evolution is to be considered the "gold standard", such as those focusing on estimating the size of minimal clinically important changes (MCIC) [32-36]. However, it requires for patients to compare their current state at the end of the study period with their recall of the initial one, which is controversial [32,33]. At the design phase, it was felt that such an approach might not be the most suitable for this study, since relying on patients' memory might have led to identifying only those changes that would have been clinically meaningful for patients, and therefore to underestimate the validity of the questionnaires that were being assessed. For that reason, in this study, patients' subjective classification of their current level of pain and disability at each assessment was used as the external criterion, and it was used to assess their matching with the scores on the NDI, NPQ and COM at that very moment (Tables 4 and 5). Consequently, to assess responsiveness to change, the change in scores of NDI, NPQ and COM from baseline to final assessment was explored for patients whose pain and disability had improved, remained unchanged or worsened according to their subjective classification at those assessments (Table 6).

For the NDI and NPQ, scores of items not applicable in one particular patient (e.g., driving or reading) are homogeneously distributed among the other dimensions. From the theoretical point of view, this might question the validity of comparisons among patients in which different dimensions are applicable. However, this is a common feature in the Oswestry Disability Index (ODI), from which both questionnaires derive, and previous studies have shown those questionnaires to be valid and reliable [3,4,14].

The representativity of the sample is not a major concern. Participants were recruited in 9 different Spanish regions representing the entire cultural and economic spectrum of the country, both in the primary care and hospital setting, and the sample included acute, subacute and chronic patients with symptoms ranging from very mild to very severe (Tables 1 and 2).

The National Spanish Academy of the Language is a multi-national agency integrated by both Castillian and Mexican experts in Spanish. It ensures that academic language, dictionaries, and semantic and grammatical rules are homogeneous throughout the Spanish speaking world. Therefore, these versions of the NDI and COM questionnaires may be used in any Spanish speaking country, although some minor finetuning may be necessary in order to adapt it to the specific terms that may be more commonly used in informal language in some specific cultural environments.

## Conclusion

In conclusion, this study shows that the Spanish versions of both NDI and COM are comprehensible and reliable, that Spanish versions of NDI, NPQ and COM are internally consistent and valid, and that it is feasible to use any of those questionnaires in routine practice. In addition, they show that NDI is the most sensitive to change and the only questionnaire to reflect patients' evolution according to their own perception. This suggests that NDI is the best option to measure NP-related disability. It requires two more minutes than the COM to fill out, but it can be answered by the patient in the waiting room without assistance.

## Competing interests

The author(s) declare that they have no competing interests.

## Authors' contributions

FMK conceived the study and participated in its design, data collection, coordination and in the drafting of the manuscript. JB, JS and SG collaborated in coordination and data collection. AR, AM, VA, JZ and AC performed the statistical analysis and participated in the drafting of the manuscript. VA also participated in the design of the study. The rest of the authors of the study collaborated in its coordination and data collection. All authors revised the design, revised the draft manuscript and read and approved its final version.

## Pre-publication history

The pre-publication history for this paper can be accessed here:



## Supplementary Material

Additional file 1 Appendix 1. Spanish version of the NDI questionnaire. The translated and validated version of the Spanish NDI questionnaire. Click here for file

Additional file 2 Appendix 2. Spanish version of the COM questionnaire. The translated and validated version of the Spanish COM questionnaire. Click here for file

Additional file 3 Bland & Altman Method (NDI and COM). The figure represents a comparison of the scores of both versions of the NDI and COM questionnaires. Click here for file
