# Supplementary figures and images for: Psychometric characteristics of the Spanish version of instruments to measure neck pain disability
Source: BMC Musculoskelet Disord. 2008 Apr 9;9:42. doi: 10.1186/1471-2474-9-42 (PMC2375887; doi:10.1186/1471-2474-9-42)

# Appendix 1. Spanish version of the NDI questionnaire.


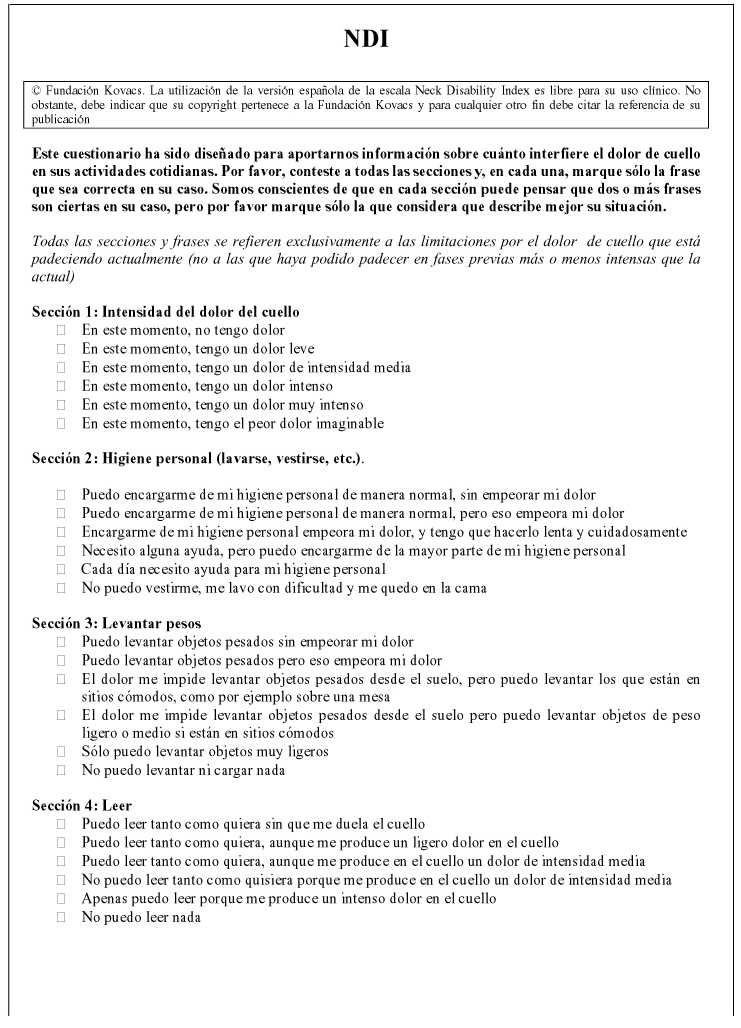


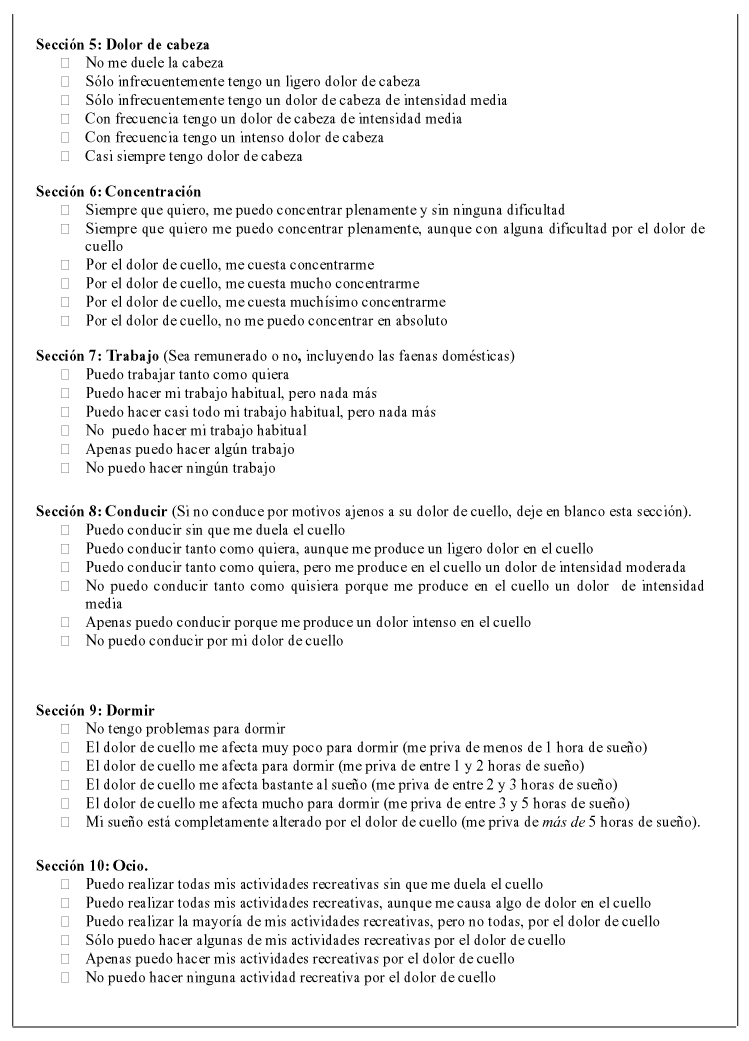

Supplement: Additional file 1 — Appendix 1. Spanish version of the NDI questionnaire. The translated and validated version of the Spanish NDI questionnaire. [file 1471-2474-9-42-S1.doc]

Appendix 2. Spanish version of the COM questionnaire.


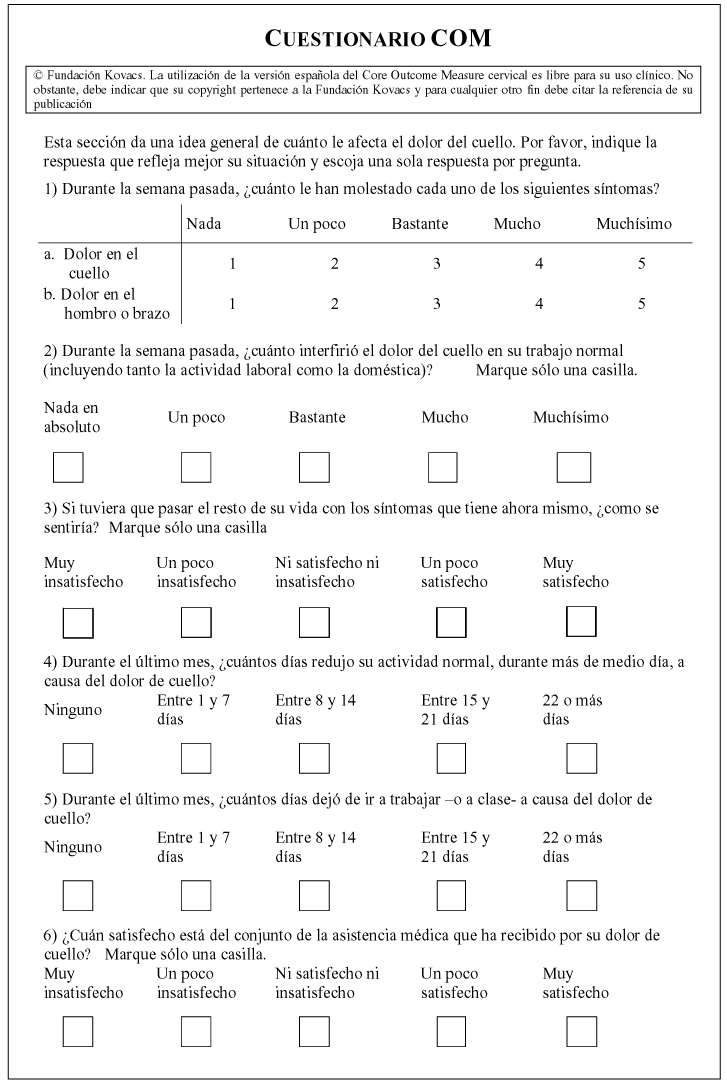

Supplement: Additional file 2 — Appendix 2. Spanish version of the COM questionnaire. The translated and validated version of the Spanish COM questionnaire. [file 1471-2474-9-42-S2.doc]

## Slide 1
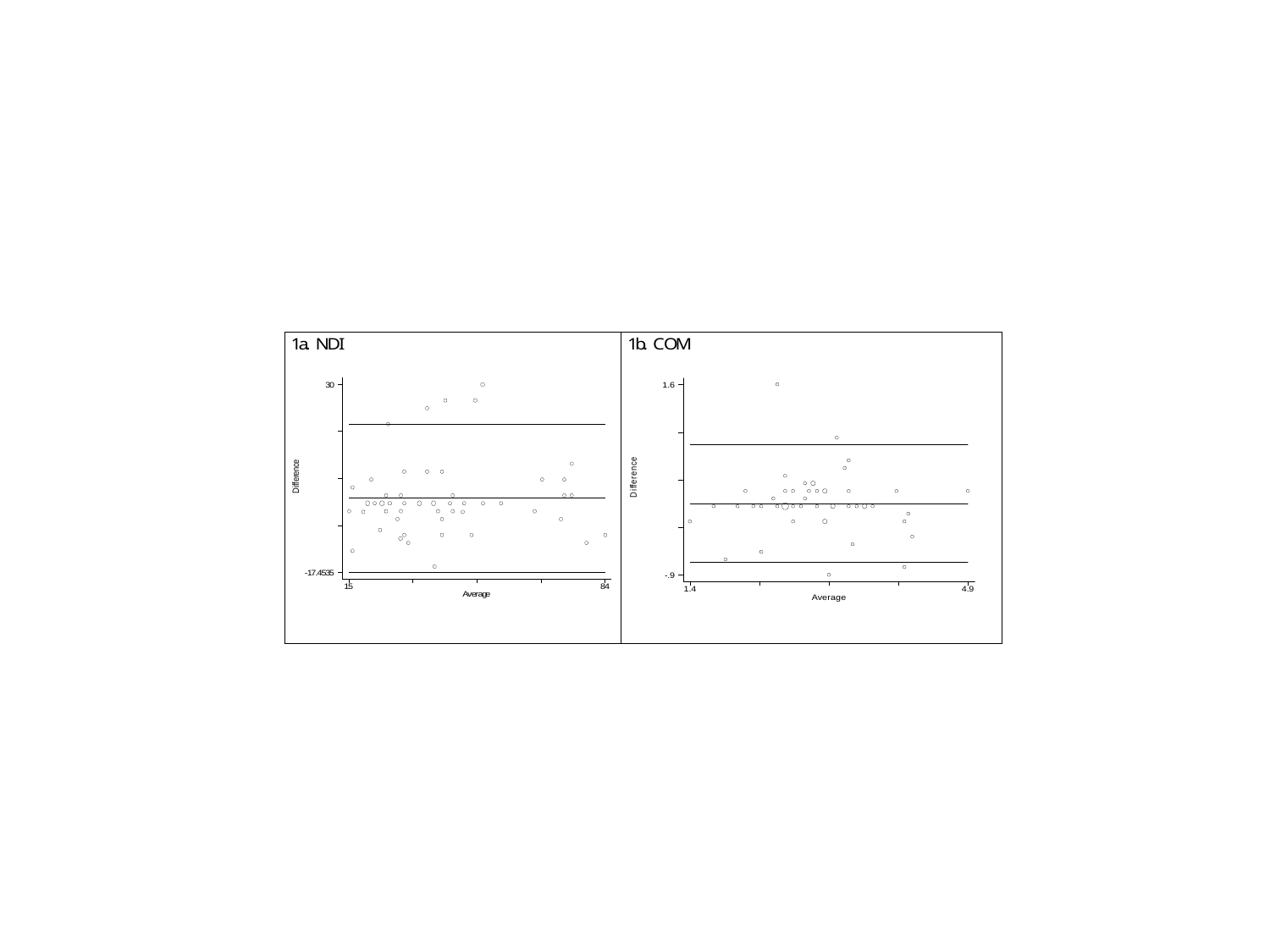

Supplement: Additional file 3 — Bland & Altman Method (NDI and COM). The figure represents a comparison of the scores of both versions of the NDI and COM questionnaires. [file 1471-2474-9-42-S3.ppt]
